# Iodine Nutrition, Non-Genetic Factors, and Two Candidate Polymorphisms Associated with Thyroid Function Abnormalities Among Children Aged 8–10 Years in Iodine-Adequate Areas of Tianjin, China

**DOI:** 10.3390/nu18183085

**Published:** 2026-09-20

**Authors:** Yani Duan, Yang Wang, Fang Li, Wenfeng Li, Dandan Zhang, Yushan Cui

**Affiliations:** Institute of Environment and Public Health, Tianjin Centers for Disease Control and Prevention, 6 Huayue Road, Hedong District, Tianjin 300011, China

**Keywords:** children, thyroid function abnormality, subclinical thyroid dysfunction, *TSHR*, *NKX2-1*, Iodine status

## Abstract

**Background/Objectives**: As dietary and environmental exposures change, interacting with genetic susceptibility, the prevalence of thyroid function abnormality and subclinical thyroid dysfunction in children is rising, yet systematic research in this population remains scarce. **Methods**: From 13 districts of Tianjin, China, all characterized by adequate iodine levels, 997 children between the ages of 8–10 years were recruited using a school-based sampling approach. Peripheral venous blood samples were drawn for genotyping analysis of two single nucleotide polymorphisms (SNPs): thyroid stimulating hormone receptor (*TSHR*) rs2268458 and NK2 homeobox 1 (*NKX2-1*) rs2076735 using the Sequenom MassARRAY platform. In addition, data on 31 non-genetic factors, including urinary iodine concentration (UIC), were collected. Restricted cubic spline (RCS) models were used to examine the non-linear associations between UIC and thyroid abnormalities. Multivariable Poisson regression was applied to identify factors associated with thyroid outcomes. **Results**: The overall prevalence of thyroid function abnormality in the study population was 17.35%, with detection rates of 3.51% for subclinical hyperthyroidism and 2.21% for subclinical hypothyroidism. After adjusting for age, sex, body mass index z-score (BMI-z-score), district of residence, and recruitment year, RCS models revealed significant U-shaped non-linear associations between UIC and both free triiodothyronine (FT_3_) abnormality (*P*_overall_ = 0.003, *P*_nonlinear_ < 0.001) and overall thyroid function abnormality (*P*_overall_ = 0.042, *P*_nonlinear_ = 0.029). In multivariable analysis, after Bonferroni correction within prespecified hypothesis families, family history of thyroid disease in first-degree relatives remained significantly associated with subclinical hyperthyroidism (adjusted *p* = 0.015), and UIC < 100 μg/L remained significantly associated with overall thyroid function abnormality (adjusted *p* = 0.036). In addition, district of residence was associated with subclinical hypothyroidism after correction (adjusted *p* = 0.009). Stratified analyses revealed potential subgroup-specific effects. Children with the *NKX2-1* rs2076735 TT + TC genotype and UIC < 100 μg/L had a higher prevalence of thyroid function abnormality compared with the reference group (UIC 100–299 μg/L) (*p* = 0.031, *PR* = 3.093, 95% *CI*: 1.108, 8.636). Frequent consumption of kelp or laver soup was associated with a higher prevalence of subclinical hyperthyroidism among children with the *NKX2-1* rs2076735 CC genotype or the *TSHR* rs2268458 TT genotype (*p* = 0.049, *PR* = 3.636, 95% *CI*: 1.006, 13.140; *p* = 0.023, *PR* = 4.814, 95% *CI*: 1.245, 18.610, respectively). Among children with the *NKX2-1* rs2076735 CC genotype, those with a family history of thyroid disease had a higher prevalence of subclinical hyperthyroidism (*p* = 0.029, *PR* = 4.114, 95% *CI*: 1.152, 14.688). After Benjamini–Hochberg false discovery rate correction, none of the subgroup-specific associations remained statistically significant (adjusted *p*-values: 0.058–0.065). **Conclusions**: The prevalence of thyroid function abnormality and subclinical thyroid dysfunction was relatively high among school-aged children in this study population from Tianjin, China. After adjustment for covariates and correction for multiple comparisons, family history of thyroid disease, UIC < 100 μg/L, and district of residence remained significantly associated with subclinical hyperthyroidism, overall thyroid function abnormality, and subclinical hypothyroidism, respectively. A U-shaped association was observed between UIC and thyroid health, highlighting the importance of maintaining adequate iodine nutrition. However, stratified analyses suggested potential subgroup-specific associations that require validation in larger, independent cohorts.

## 1. Introduction

Thyroid hormones play an indispensable role in regulating metabolism, promoting growth and development, and maintaining normal neurological function. Childhood and adolescence represent a critical window for thyroid axis development and somatic growth [1]. Even minor deviations in thyroid hormone levels during this period may impair physical growth and neurocognitive development, with potential long-term adverse effects on later health. In pediatric populations, subclinical hyperthyroidism and subclinical hypothyroidism are two relatively insidious forms of thyroid function abnormality. These conditions are relatively common but often overlooked in clinical practice due to the absence of typical symptoms and the presence of only mild biochemical deviations. Nevertheless, their long-term health implications warrant attention. In recent years, the spectrum of thyroid diseases has undergone notable changes, driven by shifting environmental exposures, modifications in dietary patterns, and the implementation of universal salt iodization policies. The prevalence of traditional disorders such as endemic goiter and overt hyperthyroidism has declined. In contrast, the prevalence of certain other thyroid conditions has increased. For example, the prevalence of subclinical hypothyroidism in the Chinese population rose from 3.21% in 1999 to 12.93% in 2017 [2]. In the United States, diagnostic and epidemiologic trends of hypothyroidism have also increased significantly; among women, prevalence rose from 14.1% in 2012 to 17.0% in 2019, while among men it increased from 4.4% to 5.9% over the same period [3]. However, most current studies in this field have focused primarily on adult populations. Systematic epidemiological investigations specifically targeting children and adolescents remain scarce. Therefore, a systematic exploration of the prevalence and factors associated with thyroid function abnormality and subclinical thyroid dysfunction in Chinese children is of public health relevance.

Iodine is an essential trace element required for thyroid hormone biosynthesis. Multiple epidemiological studies have demonstrated a close association between iodine nutritional status and thyroid function indicators in children. Some studies have reported that long-term inadequate iodine intake has been associated with endemic goiter, cretinism, and impaired intellectual development [4]. Other studies have indicated that chronic excessive iodine intake may be associated with subclinical or overt hypothyroidism [5,6]. However, no consensus has been reached regarding the association between urinary iodine levels and thyroid function abnormality or subclinical thyroid dysfunction. Further evidence from large-scale epidemiological studies is needed to address these gaps.

The pathogenesis of thyroid function abnormality and subclinical thyroid dysfunction in children is complex. Numerous studies have indicated that, in addition to iodine nutrition as a key environmental factor, environmental exposures such as ambient air pollution and chemical contaminants also play important roles in the development and progression of these conditions [7,8]. Females are known to have a higher prevalence of autoimmune thyroid diseases [9,10]. A family history of thyroid disease in first-degree relatives has been associated with higher prevalence in offspring [11,12]. Environmental organic pollutants, such as polychlorinated biphenyls and dioxins, have structures similar to thyroid hormones and have been associated with alterations in hormone synthesis, transport, metabolism, and signaling [13,14]. Heavy metals including mercury, lead, and cadmium can bind to proteins and enzymes in the thyroid gland, and have been associated with thyroid disorders [15]. Chronic psychological stress has been associated with alterations in hormone secretion through the hypothalamic–pituitary–thyroid (HPT) axis and with thyroid function abnormality [16]. Despite these findings, little research has been conducted to thoroughly examine how the above factors act together in relation to thyroid function abnormality and subclinical thyroid dysfunction among children. In response to this knowledge gap, the current study was designed to examine the associations between urinary iodine levels, as well as 30 additional non-genetic factors, and thyroid function abnormality and subclinical thyroid dysfunction.

Thyroid hormone production, release, and breakdown are under the control of multiple genes. Among them, the thyroid stimulating hormone receptor (*TSHR*) and NK2 homeobox 1 (*NKX2-1*, encoding thyroid transcription factor 1, TTF-1) play critical roles in thyroid function regulation. Although other genes—such as *TPO* (thyroid peroxidase), *TG* (thyroglobulin), and *DUOX2* (dual oxidase 2)—are also involved in thyroid hormone biosynthesis, we focused on *TSHR* and *NKX2-1* for the following reasons. First, *TSHR* serves as the main mediator through which TSH exerts its action on the thyroid gland, and candidate gene studies have repeatedly linked common variants within this gene to autoimmune thyroid diseases [17,18]. Second, TTF-1 is a master transcription factor essential for thyroid development, differentiation, and maintenance of thyroid-specific gene expression; its variants have been implicated in rare syndromic congenital hypothyroidism [19]. Third, other thyroid-related genes, such as *TPO*, are primarily linked to hormone biosynthesis defects or autoimmune responses [20,21], but their common variants have not been consistently associated with subclinical thyroid dysfunction. Given the exploratory nature of our gene–environment interaction analysis and the need to preserve statistical power with a moderate sample size, we prioritized these two biologically plausible and epidemiologically supported loci. Although previous studies have linked *TSHR* and *NKX2-1* polymorphisms to thyroid function abnormality or subclinical thyroid dysfunction, nearly all of them examined a single gene or a single exposure, and few have systematically addressed how genes and environmental factors interact. Children, however, are growing rapidly, and their thyroid function appears to respond more strongly to external exposures and other risk factors than that of adults, yet evidence from this age group remains thin. School-based studies can fill this gap: by identifying what actually affects children’s thyroid health, they provide a basis for prevention and intervention efforts that are tailored rather than generic.

In this cross-sectional investigation conducted in school settings, our objective was to identify the factors shaping thyroid function abnormality and subclinical thyroid dysfunction in children, and to further evaluate the interaction and subgroup-specific effects of two genetic loci (*TSHR* rs2268458 and *NKX2-1* rs2076735) with these influencing factors.

## 2. Materials and Methods

### 2.1. Study Population and Sampling

**Figure 1:**
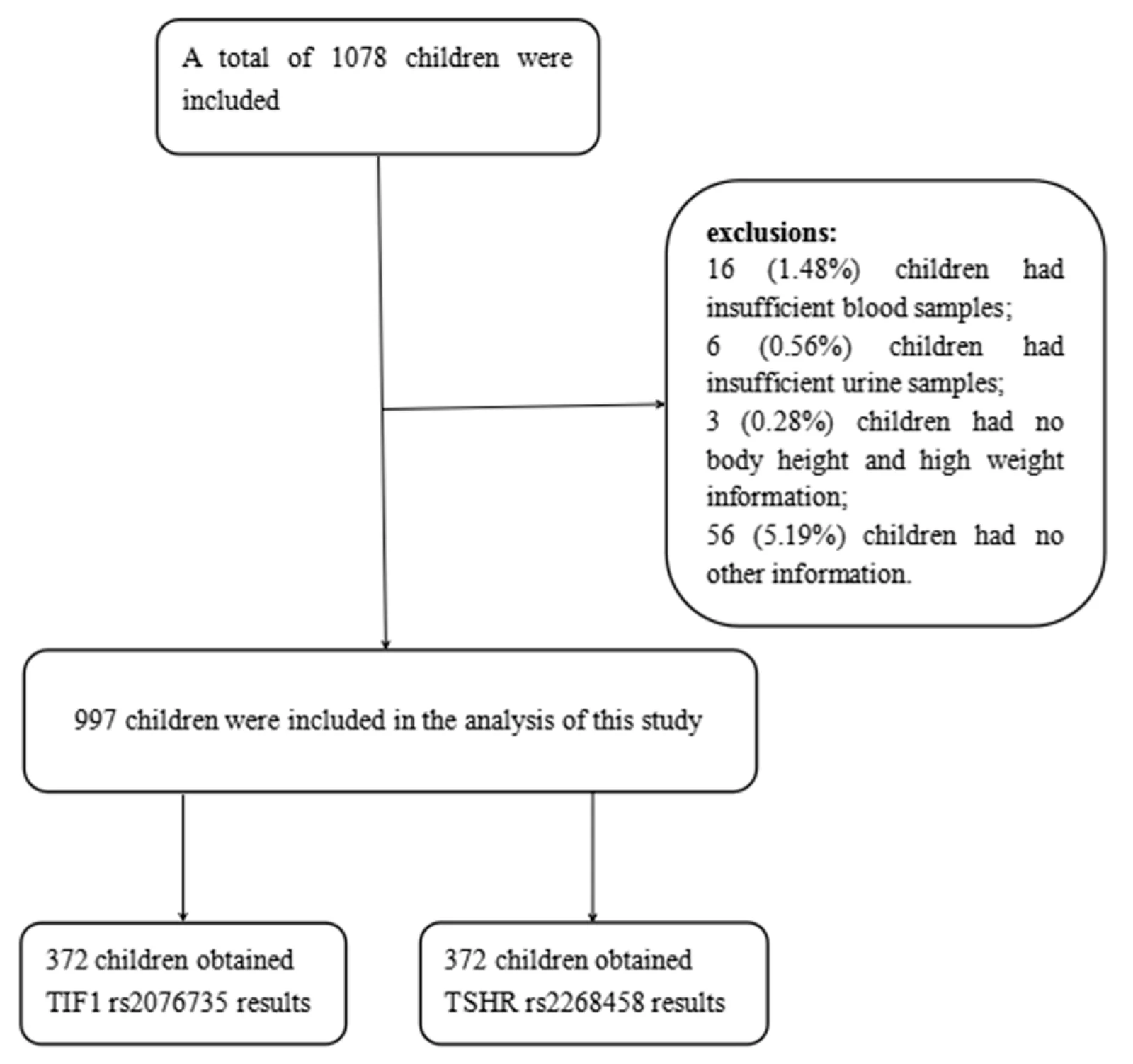
Flowchart of participant enrollment and selection.

A cross-sectional investigation was carried out among schoolchildren in Tianjin, China. According to surveillance data from the Tianjin Centers for Disease Control and Prevention (TJCDC), the 13 districts involved had each maintained adequate iodine nutrition among their monitored populations for three consecutive years. Between 2017 and 2022, one to three schools were chosen at random within each district, and children aged 8–10 years were selected through class-based cluster sampling. A total of 1078 children agreed to take part with their guardians’ consent. Participants were excluded if they (1) had incomplete questionnaire data or insufficient biological samples; (2) had endocrine, hepatic, or renal disorders or other severe conditions. After these exclusions, the final sample comprised 997 children. The flow of participants is shown in Figure 1. Written informed consent was obtained from the parents or legal guardians of all participants.

### 2.2. Anthropometry

Using standard techniques, height and weight were measured to the nearest 1 cm and 0.1 kg, respectively. Body mass index (BMI) = weight (kg)/[height (m)]^2^. To assess BMI, z-scores adjusted for age and sex were derived in accordance with the Child Growth Standards issued by the World Health Organization.

### 2.3. Urinary Iodine Assessment

All participants provided a spot urine sample, from which urinary iodine was measured. The urinary iodine concentration (UIC) was quantified through inductively coupled plasma mass spectrometry, following the Chinese standard WS/T 107.2-2016 [22]. Iodine nutrition status in children was then categorized per the criteria of the United Nations Children’s Fund (UNICEF) and the Iodine Global Network (IGN): a UIC below 100 μg/L corresponded to deficiency, 100–299 μg/L to adequate iodine nutrition, and 300 μg/L or above to excess intake [23,24].

### 2.4. Measurement of Thyroid Function and Diagnosis of Thyroid Diseases

A 3-mL sample of venous blood was drawn without anticoagulant. Serum concentrations of free triiodothyronine (FT_3_), free thyroxine (FT_4_), and thyroid-stimulating hormone (TSH) were then measured by KingMed Diagnostics (Guangzhou, China) using electrochemiluminescence immunoassay, with the Roche Cobas 8000 e602 analyzer and Roche Elecsys reagent kits (Roche Diagnostics, Mannheim, Germany).

In this study, the reference ranges for biochemical thyroid function indicators were adopted from our previous research [25], which were established based on a local pediatric population using the Roche Elecsys system (software version 06-10) to better reflect population-specific physiological characteristics. Accordingly, the following intervals were applied: TSH, 1.23–6.18 μIU/mL; FT_3_, 5.43–7.89 pmol/L; and FT_4_, 13.09–22.22 pmol/L. Values outside these ranges were defined as abnormal.

Related thyroid diseases were diagnosed according to established diagnostic criteria [26], as follows:

- Subclinical hyperthyroidism: TSH below the lower reference limit, with both FT_3_ and FT_4_ within the reference range.
- Subclinical hypothyroidism: TSH above the upper reference limit, with FT_4_ within the reference range.
- Thyroid function abnormality: at least one abnormal value among FT_3_, FT_4_, or TSH relative to the reference range. It should be noted that abnormal tests were not repeated for confirmation, and thyroid antibodies, thyroid imaging, medications, acute illness, and pubertal status were not systematically assessed. Therefore, this composite outcome represents a biochemical abnormality detected in a single screening measurement rather than clinically confirmed thyroid disease.

### 2.5. Genotyping of TSHR rs2268458 and NKX2-1 rs2076735

A 1-mL aliquot of venous blood was drawn into an anticoagulant tube, and genomic deoxyribonucleic acid (DNA) was subsequently isolated with a commercial extraction kit (Tiangen Biotech, Beijing, China). Two single nucleotide polymorphisms (SNPs)—*TSHR* rs2268458 and *NKX2-1* rs2076735—were genotyped on the Sequenom MassARRAY platform. The assay workflow comprised primer design and synthesis, assessment of DNA quality, polymerase chain reaction (PCR) amplification after primer dilution, Shrimp Alkaline Phosphatase treatment, single-base extension, resin purification, and detection by mass spectrometry. All genotyping procedures were carried out by Beijing Sequs Biotech Co., Ltd. (Beijing, China). The primers used for *TSHR* rs2268458 were as follows: first PCR primer (1st-PCRP), ACG TTG GAT GTC AAT GTG CGG GAG TTC TTC; second PCR primer (2nd-PCRP), ACG TTG GAT GAG CAC AAA GAG ACA CAC TGG. The primers for *NKX2-1* rs2076735 were as follows: 1st-PCRP, ACG TTG GAT GGA AAA CTC CCA TGC CTA CTC; 2nd-PCRP, ACG TTG GAT GCT GAT GTC GTA CAA GGA CCC.

### 2.6. Investigation of Influencing Factors

With the help of parents, questionnaire data were gathered on 31 potential influencing factors. These included age, sex, UIC, BMI, district of residence, and year of recruitment, together with 25 additional items covering three categories—lifestyle and dietary habits, health conditions, and environmental as well as socioeconomic circumstances; details of these items are provided in Table S1.

### 2.7. Statistical Methods

Continuous data were summarized as mean ± standard deviation (SD) or median (*P*_25_–*P*_75_), depending on their distribution, which was examined with the Kolmogorov–Smirnov test. Categorical variables were compared using the chi-square test, its continuity-corrected version, or Fisher’s exact test, as appropriate. To explore possible nonlinear associations of UIC with thyroid function abnormality and subclinical thyroid dysfunction, restricted cubic spline (RCS) models were fitted. Finally, Poisson regression with robust variance estimation was adopted to examine how potential influencing factors related to thyroid abnormalities; this method yields valid estimates and, for cross-sectional data with binary outcomes, is considered preferable to logistic regression [27,28]. Covariate selection was guided by a hybrid approach. Based on prior epidemiological evidence and biological plausibility, age, sex, BMI-z-score, UIC, district of residence, and recruitment year were forced into all multivariable models as prespecified confounders. Other non-genetic factors (25 in total) were initially screened using univariate Poisson regression (*p* < 0.10), and those meeting this threshold were considered for inclusion alongside the forced covariates. To account for the clustered sampling design, in which children were recruited through classes and schools across 13 districts, all multivariable Poisson regression models were fitted using generalized estimating equations (GEE) with school as the clustering unit and robust standard errors, specifying an independent working correlation matrix. For the stratified analyses, due to the limited number of events (as few as 9 cases) and low events-per-parameter ratios (2.25–6.0), covariate inclusion was determined solely by the univariate screening procedure (*p* < 0.10), applied to all non-genetic factors including age, sex, BMI, UIC, and other covariates. These results are presented as exploratory.

RCS models were fitted using logistic regression (the lrm function in the rms package of R) to evaluate the UIC–thyroid outcome association. Odds ratios (*OR*s) and 95% confidence intervals (*CI*s) were used to summarize the results. This is the standard implementation for binary outcomes in the rms package and is widely used in epidemiological studies for non-linear association analyses. We used 3 knots placed at the 10th, 50th, and 90th percentiles of the UIC distribution (13.93, 148.40, and 335.14 μg/L, respectively). The number of knots was selected based on Akaike Information Criterion (AIC); the 3-knot model consistently yielded the lowest AIC across all outcomes, providing adequate flexibility with greater parsimony compared with 4- or 5-knot models. The reference UIC value was set at the median (148.40 μg/L). Models were adjusted for age, sex, BMI-z-score, district of residence, and recruitment year. The UIC distribution in the study population had 25th, 50th, and 75th percentiles of 84.65, 148.40, and 235.10 μg/L, respectively. Overall association and nonlinearity were assessed using Wald tests (from the anova function in the rms package), and results are reported as *P*_overall_ and *P*_nonlinear_, respectively.

For influencing factors that reached significance (*p* < 0.05) in the multivariable Poisson model, additive as well as multiplicative gene–environment interactions with polymorphisms were evaluated. Poisson regression was then run separately within each genotype-defined stratum—with variables significant at *p* < 0.10 in univariate analysis included—to detect factors linked to thyroid outcomes in specific genetic subgroups. Effect estimates were expressed as prevalence ratios (*PR*s) together with their 95% *CI*s.

A prespecified two-step strategy was adopted. The first step involved testing multiplicative interactions through product terms in regression models using R, and additive interactions using the epiR package, via the relative excess risk due to interaction (*RERI*), attributable proportion (*AP*), and synergy index (*S*). The second step consisted of stratified analyses that produced genotype-specific estimates of the associations between external factors and thyroid outcomes, along with 95% *CI*s; these were examined for direction, magnitude, and precision to explore heterogeneity, but were not intended to supplant formal interaction tests [29,30].

To address the issue of multiple comparisons, we adopted a prespecified analytical framework distinguishing primary analyses from exploratory analyses. The primary hypotheses were defined as (1) the associations between UIC and thyroid abnormalities using RCS models, and (2) the associations between non-genetic factors and thyroid abnormality outcomes using multivariable Poisson regression. For the primary RCS analyses, Bonferroni correction was applied to the overall tests of the primary outcomes. For the primary Poisson regression analyses, we prespecified different independent hypothesis families based on different scientific questions (e.g., demographic and individual characteristics, and family history and socioeconomic background). All other analyses—including genetic association tests, interaction analyses, and genotype-stratified analyses—were considered exploratory and were corrected using the Benjamini–Hochberg false discovery rate method.

Sensitivity analysis was also performed with logistic regression models to confirm the robustness of the results. The same covariate selection strategies applied in the primary analyses were adopted: age, sex, BMI-z-score, UIC, district of residence, and recruitment year were forced into all multivariable models, while other covariates were screened using univariate logistic regression (*p* < 0.10). Furthermore, to account for the clustered sampling design, all multivariable logistic regression models were fitted using GEE with school as the clustering unit and robust standard errors, consistent with the primary Poisson regression analyses. Stratified logistic regression analyses were also conducted within genotype-defined subgroups to examine the associations between influencing factors and thyroid outcomes across different genetic strata, consistent with the stratification strategy used in the primary analysis. To evaluate the magnitude of the relationships between the studied variables and thyroid abnormalities, *OR*s accompanied by their corresponding 95% *CI*s were calculated.

A two-sided *p*-value of less than 0.05 was regarded as indicative of statistical significance throughout the analyses. All statistical computations were carried out with IBM SPSS Statistics version 24.0 and R software version 4.4.3.

## 3. Results

### 3.1. Participant Characteristics

The 997 children had a median UIC of 148.40 μg/L. Among them, 512 (51.35%) were boys and 485 (48.65%) were girls. The abnormality rates of FT_3_, FT_4_, and TSH were 7.82% (78/997), 4.61% (46/997), and 7.02% (70/997), respectively. The overall prevalence of thyroid function abnormality was 17.35% (173/997). The prevalence of subclinical hyperthyroidism and subclinical hypothyroidism was 3.51% (35/997) and 2.21% (22/997), respectively. The distributions of age, sex, BMI, urinary iodine, thyroid hormone levels, subclinical hyperthyroidism, and subclinical hypothyroidism are summarized in Table 1. The 25 external factors and their distributions are detailed in Table S1.

### 3.2. Associations Between Urinary Iodine and Thyroid Function Abnormality or Subclinical Thyroid Dysfunction

**Figure 2:**
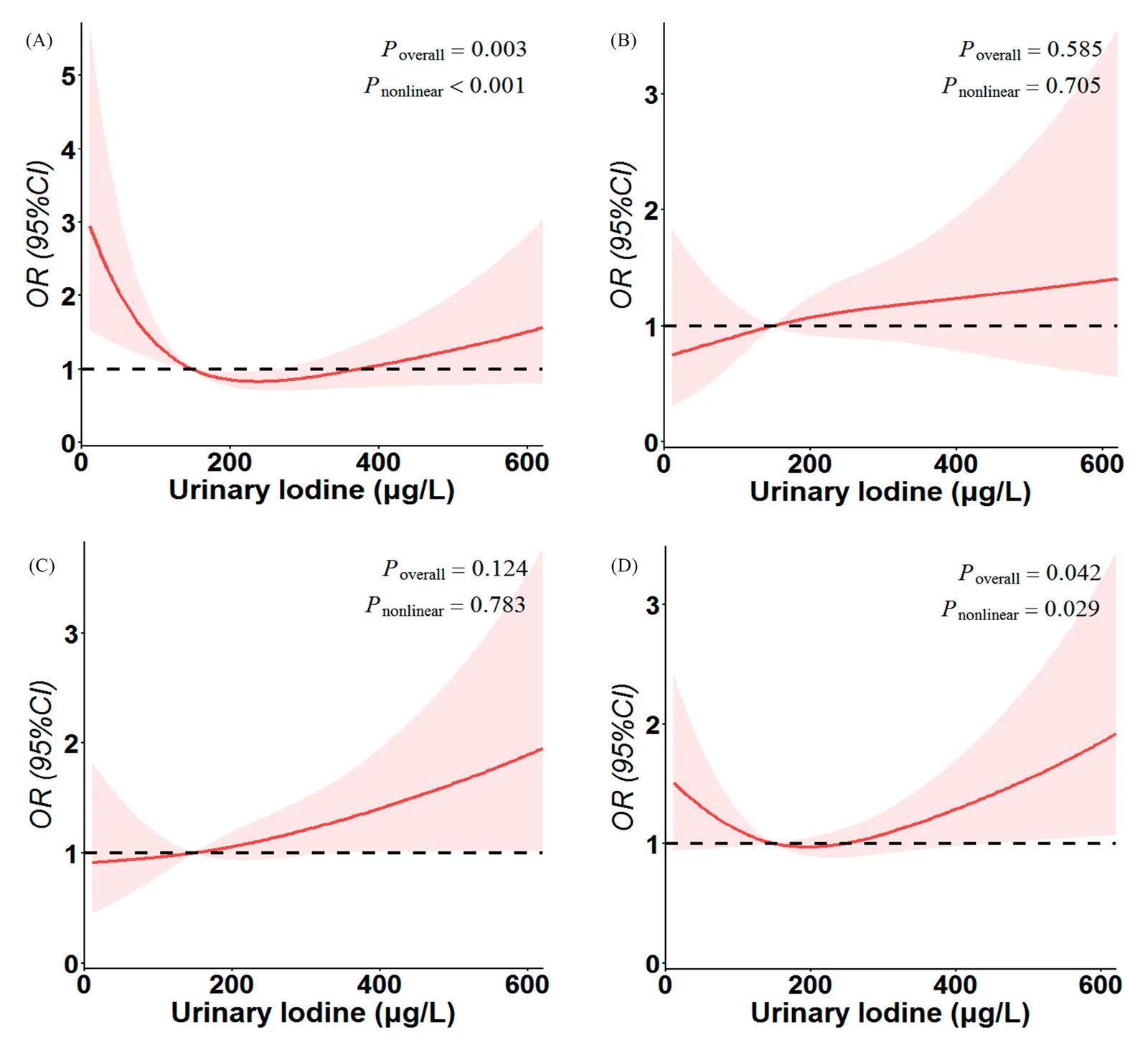
Nonlinear association of urinary iodine with thyroid function abnormalities assessed by restricted cubic splines. Note: (**A**) FT_3_ abnormality; (**B**) FT_4_ abnormality; (**C**) TSH abnormality; (**D**) composite thyroid function abnormality (defined as at least one abnormal value among FT_3_, FT_4_, or TSH relative to the reference range). The red solid line represents the fitted *OR* curve, and the red shaded area represents the 95% *CI*. The black dashed line indicates an *OR* of 1. Adjusted for covariates as follows: age, sex, BMI-z-score, district of residence, and recruitment year.

**Figure 3:**
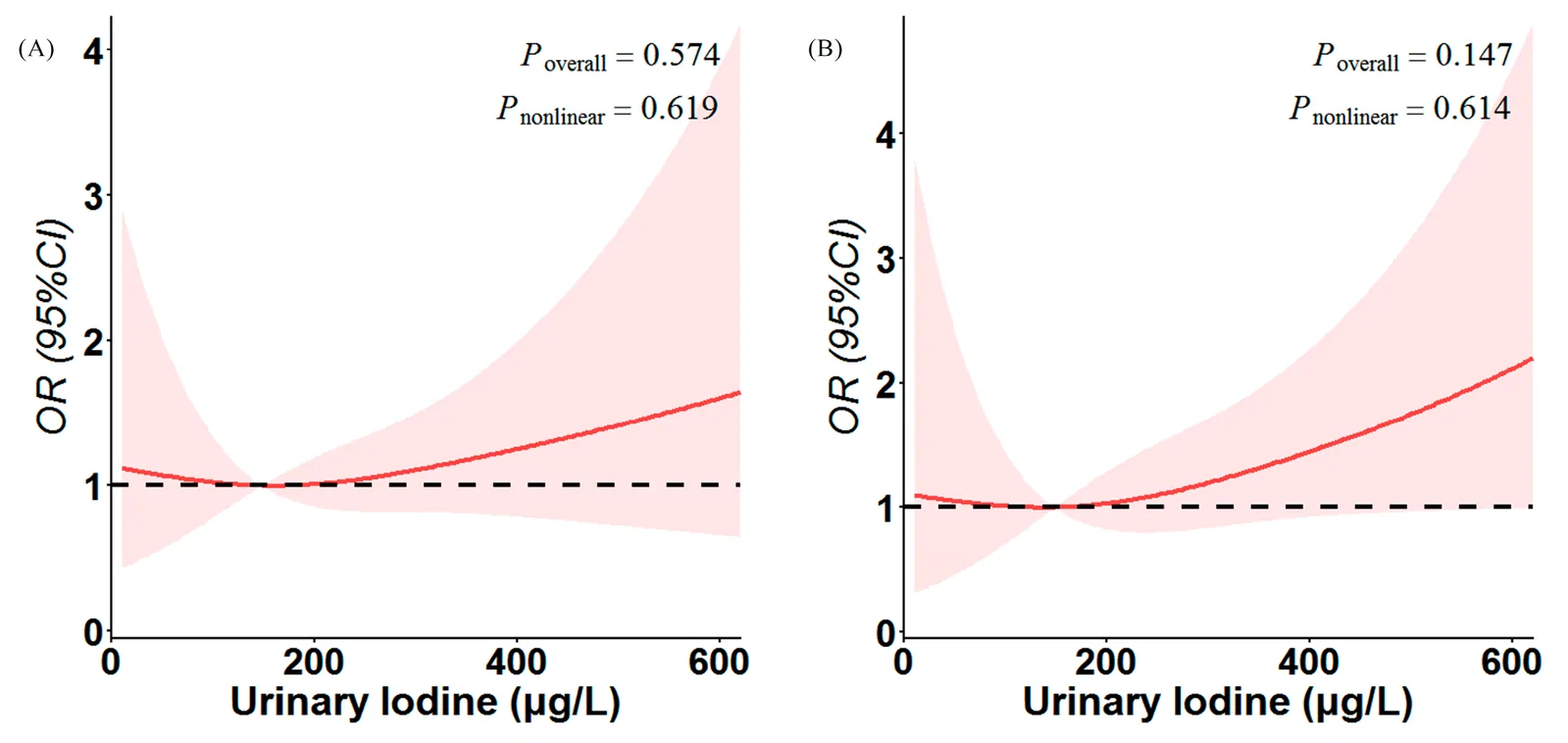
Nonlinear association of urinary iodine with subclinical thyroid dysfunction assessed by restricted cubic splines. Note: (**A**) subclinical hyperthyroidism; (**B**) subclinical hypothyroidism. The red solid line represents the fitted *OR* curve, and the red shaded area represents the 95% *CI*. The black dashed line indicates an *OR* of 1. Adjusted for covariates as follows: age, sex, BMI-z-score, district of residence, and recruitment year.

The RCS models revealed a significant U-shaped non-linear association between UIC and FT_3_ abnormality (*P*_overall_ = 0.003, *P*_nonlinear_ < 0.001), and a marginally significant U-shaped association between UIC and overall thyroid function abnormality (*P*_overall_ = 0.042, *P*_nonlinear_ = 0.029). The estimated *OR*s remained below 1.0 across UIC ranges of approximately 148.64–372.19 μg/L for FT_3_ abnormality and 148.64–246.63 μg/L for thyroid function abnormality. No significant associations were observed between UIC and FT_4_ abnormality (*P*_overall_ = 0.585), TSH abnormality (*P*_overall_ = 0.124), subclinical hyperthyroidism (*P*_overall_ = 0.574), or subclinical hypothyroidism (*P*_overall_ = 0.147). The corresponding results are presented in Figure 2 and Figure 3.

For the primary RCS analyses, the overall association between UIC and FT_3_ abnormality remained statistically significant after Bonferroni correction (*P*_overall_ = 0.003, adjusted *P*_overall_ = 0.006). The overall association between UIC and overall thyroid function abnormalities did not remain significant after Bonferroni correction (*P*_overall_ = 0.042, adjusted *P*_overal_ = 0.084).

### 3.3. Associations of Non-Genetic Factors with Thyroid Function Abnormality and Subclinical Thyroid Dysfunction

**Figure 4:**
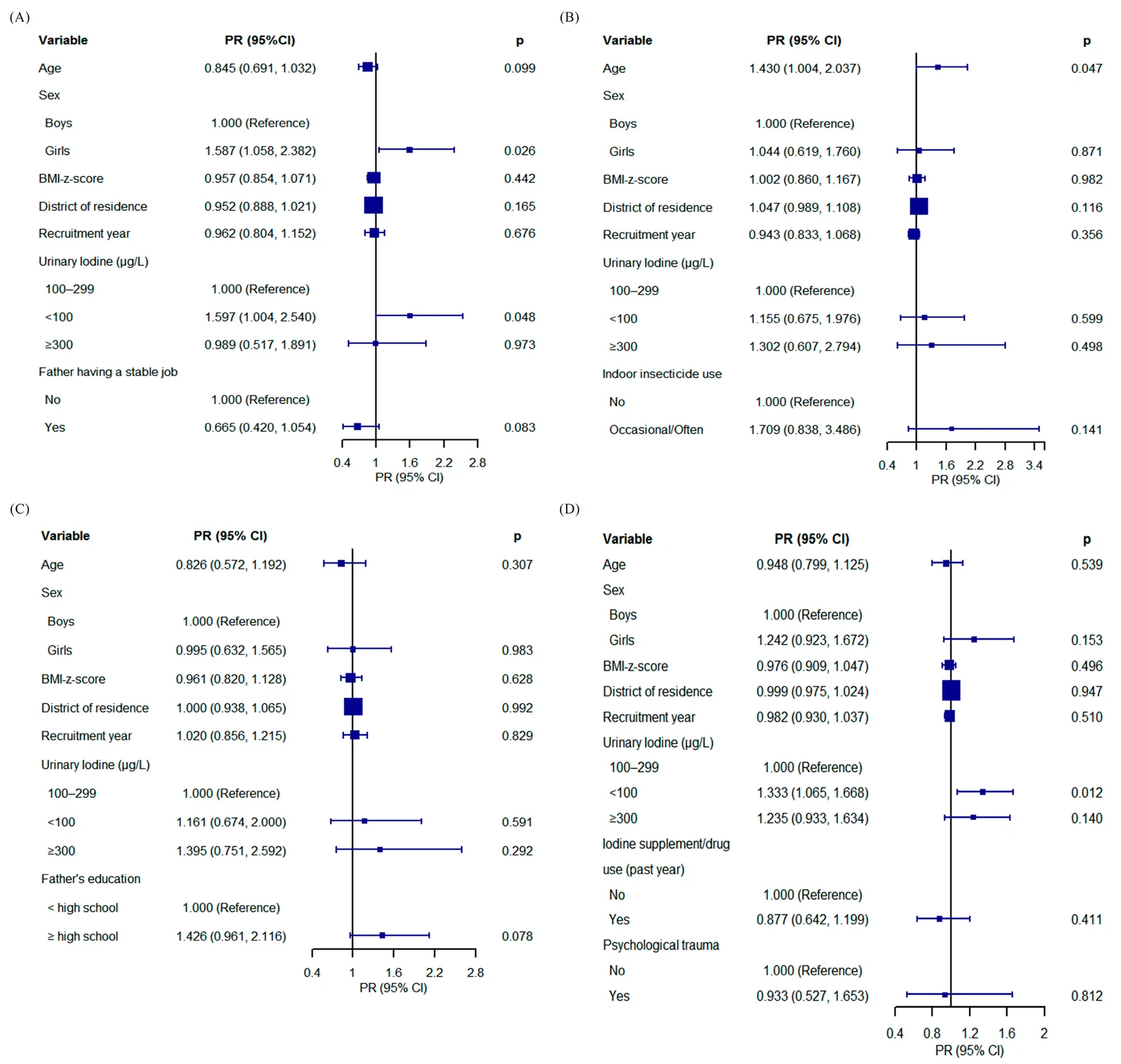
Factors associated with thyroid function abnormalities: forest plot from multivariable Poisson regression. Note: (**A**) FT_3_ abnormality; (**B**) FT_4_ abnormality; (**C**) TSH abnormality; (**D**) composite thyroid function abnormality (defined as at least one abnormal value among FT_3_, FT_4_, or TSH relative to the reference range).

**Figure 5:**
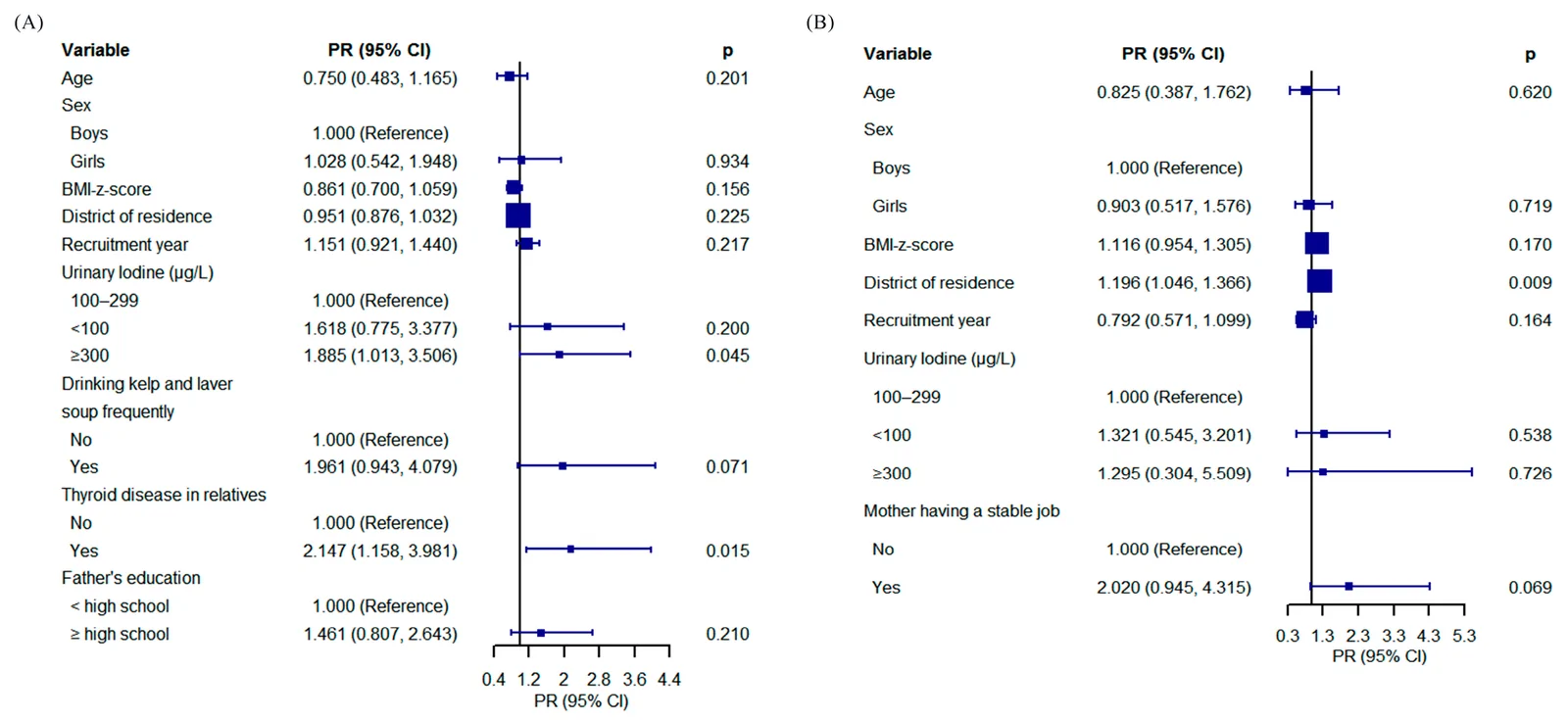
Factors associated with subclinical thyroid dysfunction: forest plot from multivariable Poisson regression. Note: (**A**) subclinical hyperthyroidism; (**B**) subclinical hypothyroidism.

Each non-genetic factor was examined in univariate analyses for its relationship with thyroid function abnormality and subclinical thyroid dysfunction in children. Factors with *p* < 0.1 are summarized in Table S2. Variables with *p* < 0.1 in univariate analyses were then included in multivariable Poisson regression models. In addition, age, sex, BMI-z-score, district of residence, recruitment year, and urinary iodine were forced into all multivariable models as prespecified confounders based on prior epidemiological evidence. The full multivariable analysis results appear in Table 2 and Figure 4 and Figure 5. To check for multicollinearity, the variance inflation factor (VIF) was calculated; all values for the included variables were under 5.0, indicating no significant multicollinearity (Table S3). The multivariable results showed that girls had a higher prevalence of FT_3_ abnormality (*p* = 0.026, *PR* = 1.587, 95% *CI*: 1.058, 2.382). UIC < 100 μg/L was associated with a higher prevalence of FT_3_ abnormality (*p* = 0.048, *PR* = 1.597, 95% *CI*: 1.004, 2.540) and overall thyroid function abnormality (*p* = 0.012, *PR* = 1.333, 95% *CI*: 1.065, 1.668). Age was associated with FT_4_ abnormality (*p* = 0.047, *PR* = 1.430, 95% *CI*: 1.004, 2.037). A family history of thyroid disease in first-degree relatives was associated with a higher prevalence of subclinical hyperthyroidism (*p* = 0.015, *PR* = 2.147, 95% *CI*: 1.158, 3.981). UIC ≥ 300 μg/L was associated with subclinical hyperthyroidism (*p* = 0.045, *PR* = 1.885, 95% *CI*: 1.013, 3.506). District of residence was associated with subclinical hypothyroidism (*p* = 0.009, *PR* = 1.196, 95% *CI*: 1.046, 1.366). For multiplicity control, we applied Bonferroni correction within prespecified hypothesis families. Within the demographic characteristics family (sex and age; 2 tests), neither sex (*p* = 0.026, adjusted *p* = 0.052) nor age (*p* = 0.047, adjusted *p* = 0.094) remained significant. Within the iodine nutrition family (UIC < 100 μg/L with FT_3_ abnormality, UIC < 100 μg/L with thyroid function abnormality, and UIC ≥ 300 μg/L with subclinical hyperthyroidism; 3 tests), UIC < 100 μg/L remained significantly associated with overall thyroid function abnormality after Bonferroni correction (adjusted *p* = 0.036), while the associations with FT_3_ abnormality (adjusted *p* = 0.144) and UIC ≥ 300 μg/L with subclinical hyperthyroidism (adjusted *p* = 0.135) did not reach statistical significance. A family history of thyroid disease in first-degree relatives remained significantly associated with subclinical hyperthyroidism after correction (*p* = 0.015, adjusted *p* = 0.015, with 1 test in the family history family). District of residence remained significantly associated with subclinical hypothyroidism after correction (*p* = 0.009, adjusted *p* = 0.009, with 1 test in the geographic factor family).

### 3.4. Associations of Genotypes with Thyroid Function Abnormality and Subclinical Thyroid Dysfunction

The associations of *TSHR* rs2268458 and *NKX2-1* rs2076735 polymorphisms with thyroid function abnormality and subclinical thyroid dysfunction were examined in children. Genotyping was performed on a subset of the study population. Specifically, 372 children were randomly selected from several districts for genotyping, representing a subcohort of the full study population of 997 participants. The genotyping call rate was 100% for both SNPs in this subset, with no missing genotype data observed. Minor allele frequencies (MAF) were 0.164 for *NKX2-1* rs2076735 and 0.269 for *TSHR* rs2268458. Hardy–Weinberg equilibrium was tested for both polymorphisms as a quality control measure for genotyping accuracy. The genotype distributions of both loci were in accordance with Hardy–Weinberg equilibrium (*NKX2-1* rs2076735: *p* = 0.449; *TSHR* rs2268458: *p* = 0.576), indicating no significant deviation from expected genotype frequencies. The detailed results are presented in Table S4. Three genetic models were applied to rs2268458 (CC vs. CT vs. TT; CC vs. CT + TT; CC + CT vs. TT) and to rs2076735 (CC vs. TC vs. TT; CC vs. TC + TT; CC + TC vs. TT). The distribution of thyroid function abnormality and subclinical thyroid dysfunction across different genotype groups was compared under each model. A significant difference was observed in the distribution of subclinical hyperthyroidism among children carrying different rs2268458 genotypes under the codominant model (CC vs. CT vs. TT) (*p* < 0.05). However, after chi-square partition, no significant differences were found between any two genotype groups (all *p* > 0.0167). No significant associations were found between the other genetic models and thyroid function abnormality or subclinical thyroid dysfunction (all *p* > 0.05). The detailed results are presented in Table S5.

### 3.5. Multiplicative and Additive Interactions of TSHR rs2268458 and NKX2-1 rs2076735 with Influencing Factors on Thyroid Function Abnormality and Subclinical Thyroid Dysfunction

To assess the joint effects of genetic variants and influencing factors (those reaching *p* < 0.05 in the multivariable Poisson regression) on thyroid function abnormality and subclinical thyroid dysfunction, both multiplicative and additive interaction analyses were conducted. The additive interaction results are summarized in Table 3: neither *TSHR* rs2268458 nor *NKX2-1* rs2076735 exhibited a statistically significant additive interaction with any influencing factor. Likewise, the multiplicative interaction models revealed no statistically significant interaction effects (Table 4).

### 3.6. Genotype-Stratified Associations of Influencing Factors with Thyroid Function Abnormality and Subclinical Thyroid Dysfunction

We further performed stratified analyses by genotype using multivariable Poisson regression models. Factors with *p* < 0.1 in univariate analyses were included to explore their associations with thyroid function abnormality and subclinical thyroid dysfunction within each genotype subgroup. For *TSHR* rs2268458, participants were categorized into two groups: CC + CT versus TT. For *NKX2-1* rs2076735, participants were grouped as CC versus TT + TC. We used these stratifications to evaluate the potential subgroup-specific effects of each polymorphism.

As shown in Table 5, in stratified analyses, some subgroup-specific associations were observed, although formal additive and multiplicative interaction tests did not reach statistical significance (Table 3 and Table 4). For instance, among children carrying the *NKX2-1* rs2076735 TT + TC genotype, those with UIC < 100 μg/L had a higher prevalence of thyroid function abnormality compared with the reference group (UIC 100–299 μg/L) (*p* = 0.031, *PR* = 3.093, 95% *CI*: 1.108, 8.636). Similarly, among children with the *NKX2-1* rs2076735 CC genotype or the *TSHR* rs2268458 TT genotype, frequent consumption of kelp or laver soup was associated with a higher prevalence of subclinical hyperthyroidism (*p* = 0.049, *PR* = 3.636, 95% *CI*: 1.006, 13.140; *p* = 0.023, *PR* = 4.814, 95% *CI*: 1.245, 18.610, respectively). In addition, among children with the *NKX2-1* rs2076735 CC genotype, those with a family history of thyroid disease in first-degree relatives showed a higher prevalence of subclinical hyperthyroidism (*p* = 0.029, *PR* = 4.114, 95% *CI*: 1.152, 14.688). However, after applying Benjamini–Hochberg false discovery rate correction for multiple comparisons, none of these associations remained statistically significant (adjusted *p*-values: 0.058–0.065). In addition, the events per parameter ratios ranged from 2.25 to 6.0 (Table S6).

### 3.7. Sensitivity Analysis

Factors with *p* < 0.1 are summarized in Table S7. To further verify the robustness of the Poisson regression results, a sensitivity analysis was conducted using logistic regression with the same covariate selection and clustering adjustment strategies as the primary analyses. The main findings remained largely consistent with the primary results (Table S8).

Logistic regression was also used to explore the subgroup-specific effects of genotypes on the associations between influencing factors and thyroid function abnormality or subclinical thyroid dysfunction. The results were consistent with the primary stratified analyses (Table S9).

## 4. Discussion

In this study, the overall prevalence of thyroid function abnormality was 17.35%, with detection rates of 3.51% for subclinical hyperthyroidism and 2.21% for subclinical hypothyroidism. In a cross-sectional study of Chinese children, the reported detection rates were 2.48% for subclinical hyperthyroidism and 4.88% for subclinical hypothyroidism [31]. In other pediatric populations, the reported detection rate for subclinical hypothyroidism was 22.80% among Iranian children aged 2–10 years [32] and 11.79% in an Indian study [33]. It should be noted that these prevalence estimates may not be directly comparable due to variations in age groups, sampling strategies, reference intervals, and outcome definitions across studies; the comparisons presented here are intended to provide general context rather than to establish direct epidemiological contrasts. The detection rates of subclinical hypothyroidism were lower than those reported in Iran and India. Such discrepancies could stem from multiple sources, such as regional eating habits, inherited susceptibility, and exposure to environmental pollutants. Nevertheless, the relatively high detection rate of thyroid function abnormality in children warrants attention. Because subclinical hyperthyroidism and subclinical hypothyroidism present without obvious clinical symptoms, awareness of these conditions among parents and primary healthcare providers remains limited. Prolonged untreated conditions may have implications for neurodevelopment and physical growth in children. Given the insidious impact of thyroid function abnormality and subclinical thyroid dysfunction on neurodevelopment and physical growth during childhood, further research is warranted to clarify the clinical implications of these biochemical abnormalities.

Iodine nutrition is a key determinant of thyroid function, and its relationship with thyroid disorders has been extensively investigated. However, findings from previous studies remain inconsistent. One cross-sectional study reported no significant association between iodine status and the detection rates of thyroid disorders or the positivity rates of thyroid autoantibodies [34]. In contrast, a nationwide cross-sectional study involving 78,470 participants across 31 provincial-level regions in mainland China found that iodine excess was only associated with higher odds of overt hyperthyroidism and subclinical hypothyroidism, whereas iodine deficiency was significantly associated with higher odds of most thyroid disorders. In addition, increased iodine intake was significantly correlated with elevated serum TSH levels [2]. In the present study, RCS models revealed a significant non-linear association between UIC and FT_3_ abnormality, showing a U-shaped pattern. The estimated *OR* remained below 1.0 across a UIC range from approximately 148.64 to 372.19 μg/L. Similarly, a significant non-linear U-shaped association was observed between UIC and overall thyroid function abnormality, with the estimated *OR* below 1.0 across a UIC range from approximately 148.64 to 246.63 μg/L. These results are largely consistent with reports describing a U-shaped or J-shaped relationship between iodine intake and thyroid function abnormality [2,35]. These curve-derived intervals are data-dependent and should not be interpreted as validated clinical thresholds for optimal iodine status. Inadequate iodine intake impairs thyroid hormone synthesis, leading to compensatory TSH elevation, which over time may contribute to thyroid hyperplasia and functional decompensation. Conversely, excessive iodine exposure can transiently inhibit iodine organification (the Wolff–Chaikoff effect), and in susceptible individuals, sustained iodine excess may lead to abnormal thyroid hormone secretion [36]. High iodine has also been associated with alterations in thyroid autoimmunity, which may contribute to thyroid dysfunction. The U-shaped association observed in our study underscores the importance of maintaining adequate iodine status for thyroid health in children.

However, no significant associations were observed between UIC and FT_4_ abnormality, TSH abnormality, subclinical hyperthyroidism, or subclinical hypothyroidism in this study. Several factors may explain these null findings. First, FT_4_ levels are regulated by thyroid hormone-binding proteins and peripheral conversion, which may affect its responsiveness to nutritional and metabolic stimuli. Second, TSH secreted by the pituitary gland may exhibit a delayed response to thyroidal perturbations. Third, the pathogenesis of subclinical thyroid dysfunction is more complex and involves not only iodine nutrition but also autoimmune, genetic, and environmental factors [37,38,39]. In addition, the study population generally had adequate iodine status, with relatively low proportions of extreme iodine excess or severe deficiency, which may have reduced the strength of the associations between UIC and subclinical conditions.

In this study, univariate and multivariable Poisson regression analyses were performed to identify factors associated with thyroid abnormalities. After Bonferroni correction for multiple comparisons within prespecified hypothesis families, family history of thyroid disease in first-degree relatives remained significantly associated with subclinical hyperthyroidism, which is consistent with the view that thyroid diseases have a familial aggregation and a genetic predisposition [11,40]. In addition, UIC < 100 μg/L remained significantly associated with overall thyroid function abnormality, further supporting the U-shaped association observed in the RCS analyses. District of residence was also associated with subclinical hypothyroidism after correction; however, as district was treated as a continuous variable to preserve degrees of freedom, this finding should be interpreted with caution and may reflect unmeasured regional differences in iodine nutrition, dietary patterns, or healthcare access. Thyroid disorders are considered to have a complex etiology involving both genetic and environmental factors. The presence of affected family members may be associated with higher prevalence in offspring through multiple pathways, including genetic inheritance, shared living environments, and similar dietary habits.

*TSHR* and *NKX2-1* are key genes involved in thyroid development and function regulation. *TSHR* encodes the thyroid stimulating hormone receptor, which mediates the regulatory effects of TSH on the thyroid gland. Genetic variants in *TSHR* have been associated with receptor function and have been linked to abnormal thyroid hormone secretion [41]. TTF-1 acts as a transcription factor and participates in thyroid cell differentiation and thyroid hormone synthesis. Polymorphisms in the *NKX2-1* gene have been closely linked to goiter and congenital thyroid function abnormality [42].

In the present study, the polymorphisms of *TSHR* rs2268458 and *NKX2-1* rs2076735 did not appear to have strong independent main effects in children. This finding is not entirely consistent with some previous reports. For instance, Yin X et al. [43] found that the rs2268458 C allele was significantly associated with Graves’ disease in Caucasian women. A study from Iran reported that the rs2268458 C allele was associated with both hypothyroidism and hyperthyroidism [44]. Several reasons may explain these discrepancies. First, the study population consisted of children, and the pathogenic mechanisms of thyroid function abnormality in children may differ from those in adults. Thyroid function abnormality in children is more often related to iodine nutrition and developmental factors, whereas autoimmune thyroid diseases are more dominant in adults. Second, the type and severity of thyroid diseases in our study population may differ from those in previous studies, with a potentially lower proportion of autoimmune thyroiditis. Third, the frequency distribution and effect sizes of genetic polymorphisms may vary across different ethnic and geographic populations. It is worth noting that although no significant associations were found between the two SNP loci and thyroid function abnormality or subclinical thyroid dysfunction, this does not imply that these polymorphisms are not involved in thyroid developmental abnormalities.

Multiplicative and additive models were applied to examine interactions between influencing factors and two polymorphisms, *TSHR* rs2268458 and *NKX2-1* rs2076735 [45,46]. Neither model yielded evidence of interaction between the two polymorphisms and the factors examined. This held true across all thyroid outcomes assessed, including thyroid function abnormality and subclinical thyroid dysfunction. It is important to distinguish between statistical association and biological interaction. Our analyses identified statistical associations between certain environmental factors and thyroid outcomes, and we tested for statistical interactions on both additive and multiplicative scales. However, the lack of significant interaction tests indicates that we cannot conclude biological effect modification by genotype. The observed subgroup-specific findings represent statistical associations that may be influenced by limited statistical power and multiple testing, and may not represent evidence of true biological interaction. The stratified analyses revealed some subgroup-specific associations that merit further investigation, although these findings should be interpreted with caution. For instance, among children carrying the *NKX2-1* rs2076735 TT + TC genotype, those with UIC < 100 μg/L had a higher prevalence of thyroid function abnormality compared with the reference group (UIC 100–299 μg/L). Among children carrying the CC genotype at *NKX2-1* rs2076735 or the TT genotype at *TSHR* rs2268458, frequent consumption of kelp or laver soup was associated with a higher prevalence of subclinical hyperthyroidism in the stratified analyses. In addition, among children with the *NKX2-1* rs2076735 CC genotype, a family history of thyroid disease in first-degree relatives was also associated with subclinical hyperthyroidism. However, after applying Benjamini–Hochberg false discovery rate correction for multiple comparisons within this exploratory analysis family, none of these associations remained statistically significant (adjusted *p*-values ranged from 0.058 to 0.065), suggesting that the original borderline findings (*p* = 0.023–0.049) may reflect chance. These results should be considered exploratory and hypothesis-generating. Future studies with larger sample sizes and adequate power to detect gene–environment interactions are needed to validate these observations.

This study has several notable strengths. First, methodologically, we used Poisson regression models to estimate *PR* rather than *OR*, which is more appropriate for cross-sectional studies as *PR* more accurately reflects the strength of associations between exposures and outcomes. In addition, the application of RCS models enabled the flexible examination of non-linear associations between UIC and thyroid abnormalities, avoiding information loss that may result from simple linear assumptions. Second, to assess how genetic and non-genetic factors jointly affect thyroid health in children, we incorporated a broad set of variables. These comprised two key polymorphisms, *TSHR* rs2268458 and *NKX2-1* rs2076735, together with 31 factors linked to child development. This comprehensive approach strengthened our capacity to capture diverse potential determinants of thyroid health. In addition, we examined not only gene–environment interactions but also the subgroup-specific influence of genetic background on these associations. However, several limitations should be acknowledged. The cross-sectional design of this study precludes causal inferences. We only examined two genetic loci, *TSHR* rs2268458 and *NKX2-1* rs2076735, and the associations of other thyroid disease-related genes were not explored. Thyroid peroxidase antibody (TPOAb) and thyroglobulin antibody (TgAb) were not measured in this study. Autoimmune thyroiditis is a common cause of thyroid function abnormality, and the absence of these biomarkers prevents us from excluding underlying autoimmune processes as a potential confounder in the observed associations. This study was conducted exclusively among school-aged children from 13 districts in Tianjin, China, so the results may not be generalizable to other populations. Finally, iodine nutrition was assessed using a single spot UIC, which has high within-person variability and may not reflect habitual intake. This limitation likely introduces non-differential misclassification in gene-environment analyses, biasing associations toward the null. Therefore, these exploratory findings require validation in future studies using repeated UIC or 24-h urine collections.

## 5. Conclusions

This study, conducted among 997 children, found that the overall prevalence of thyroid function abnormality was 17.35%, with detectable proportions of subclinical hyperthyroidism and subclinical hypothyroidism. These findings highlight the need for attention to thyroid health in children. After adjusting for covariates, UIC showed a U-shaped non-linear association with FT_3_ abnormality and overall thyroid function abnormality. In multivariable analyses, after Bonferroni correction for multiple comparisons, family history of thyroid disease in first-degree relatives, UIC < 100 μg/L, and district of residence remained significantly associated with subclinical hyperthyroidism, overall thyroid function abnormality, and subclinical hypothyroidism, respectively. Stratified analyses suggested potential subgroup-specific associations that require validation in larger, independent cohorts. Given the cross-sectional design, these findings highlight the need for prospective cohort studies to confirm the observed associations.

## Figures and Tables

**Table 1:** Characteristics of the study participants.

Characteristics	*n*	Statistics
Age (years)	997	9.00 (9.00–10.00)
Sex		
Boys	512	51.35
Girls	485	48.65
BMI (kg/m^2^)	997	17.97 ± 3.73
BMI-z-score	997	0.51 ± 1.57
Urinary Iodine (μg/L)	997	148.40 (84.65–235.10)
FT_3_ (pmol/L)	997	6.55 (6.07–7.00)
FT_4_ (pmol/L)	997	17.28 (15.95–18.80)
TSH (μIU/mL)	997	2.74 (2.05–3.69)
FT_3_ abnormality	78	7.82
FT_4_ abnormality	46	4.61
TSH abnormality	70	7.02
Thyroid function abnormality	173	17.35
Subclinical hyperthyroidism	35	3.51
Subclinical hypothyroidism	22	2.21

Note: Data are presented as %, mean ± SD, or median (*P*_25_, *P*_75_).

**Table 2:** Thyroid function abnormalities and subclinical thyroid dysfunction: influencing factors in multivariable-adjusted Poisson regression.

Thyroid Outcomes	Associated Factors	*p*	*PR* (95% *CI*)
FT_3_ abnormality	Age	0.099	0.845 (0.691, 1.032)
	Sex	0.026	1.587 (1.058, 2.382)
	BMI-z-score	0.442	0.957 (0.854, 1.071)
	District of residence	0.165	0.952 (0.888, 1.021)
	Recruitment year	0.676	0.962 (0.804, 1.152)
	Urinary Iodine		
	<100 μg/L	0.048	1.597 (1.004, 2.540)
	≥300 μg/L	0.973	0.989 (0.517, 1.891)
	Father having a stable job	0.083	0.665 (0.420, 1.054)
FT_4_ abnormality	Age	0.047	1.430 (1.004, 2.037)
	Sex	0.871	1.044 (0.619, 1.760)
	BMI-z-score	0.982	1.002 (0.860, 1.167)
	District of residence	0.116	1.047 (0.989, 1.108)
	Recruitment year	0.356	0.943 (0.833, 1.068)
	Urinary Iodine		
	<100 μg/L	0.599	1.155 (0.675, 1.976)
	≥300 μg/L	0.498	1.302 (0.607, 2.794)
	Indoor insecticide use	0.141	1.709 (0.838, 3.486)
TSH abnormality	Age	0.307	0.826 (0.572, 1.192)
	Sex	0.983	0.995 (0.632, 1.565)
	BMI-z-score	0.628	0.961 (0.820, 1.128)
	District of residence	0.992	1.000 (0.938, 1.065)
	Recruitment year	0.829	1.020 (0.856, 1.215)
	Urinary Iodine		
	<100 μg/L	0.591	1.161 (0.674, 2.000)
	≥300 μg/L	0.292	1.395 (0.751, 2.592)
	Father’s education	0.078	1.426 (0.961, 2.116)
Thyroid function abnormality	Age	0.539	0.948 (0.799, 1.125)
	Sex	0.153	1.242 (0.923, 1.672)
	BMI-z-score	0.496	0.976 (0.909, 1.047)
	District of residence	0.947	0.999 (0.975, 1.024)
	Recruitment year	0.510	0.982 (0.930, 1.037)
	Urinary Iodine		
	<100 μg/L	0.012	1.333 (1.065, 1.668)
	≥300 μg/L	0.140	1.235 (0.933, 1.634)
	Iodine supplement/drug use (past year)	0.411	0.877 (0.642, 1.199)
	Psychological trauma	0.812	0.933 (0.527, 1.653)
Subclinical hyperthyroidism	Age	0.201	0.750 (0.483, 1.165)
	Sex	0.934	1.028 (0.542, 1.948)
	BMI-z-score	0.156	0.861 (0.700, 1.059)
	District of residence	0.225	0.951 (0.876, 1.032)
	Recruitment year	0.217	1.151 (0.921, 1.440)
	Urinary Iodine		
	<100 μg/L	0.200	1.618 (0.775, 3.377)
	≥300 μg/L	0.045	1.885 (1.013, 3.506)
	Drinking kelp and laver soup frequently	0.071	1.961 (0.943, 4.079)
	Thyroid disease in relatives	0.015	2.147 (1.158, 3.981)
	Father’s education	0.210	1.461 (0.807, 2.643)
Subclinical hypothyroidism	Age	0.620	0.825 (0.387, 1.762)
	Sex	0.719	0.903 (0.517, 1.576)
	BMI-z-score	0.170	1.116 (0.954, 1.305)
	District of residence	0.009	1.196 (1.046, 1.366)
	Recruitment year	0.164	0.792 (0.571, 1.099)
	Urinary Iodine		
	<100 μg/L	0.538	1.321 (0.545, 3.201)
	≥300 μg/L	0.726	1.295 (0.304, 5.509)
	Mother having a stable job	0.069	2.020 (0.945, 4.315)

Note: Reference: urinary iodine 100–299 μg/L. District of residence was treated as a continuous variable (coded 1–13) in the multivariable models to preserve degrees of freedom, given the limited number of events in certain outcome strata. This approach was applied only to adjust for geographic variation rather than to estimate district-specific effects.

**Table 3:** Thyroid function abnormalities and subclinical thyroid dysfunction: additive interaction of SNPs with influencing factors.

		Additive Model		
		*RERI* (95% *CI*)	*AP* (95% *CI*)	*S* (95% *CI*)
FT_3_ abnormality				
	Sex and rs2268458	−1.412 (−18.528,1.134)	−1.022 (−12.771, 0.785)	0.214 (−3.455,3.272)
	Sex and rs2076735	−0.189 (−8.847, 1.268)	−0.225 (−5.633, 2.444)	−5.884 (−3.214, 4.309)
	Urinary iodine and rs2268458	0.778 (−0.845, 1.807)	1.167 (−1.297, 4.513)	0.300 (−2.091, 2.809)
	Urinary iodine and rs2076735	0.580 (−1.279, 1.143)	1.891 (−2.500, 9.524)	0.545 (−0.836, 2.110)
FT_4_ abnormality				
	Age and rs2268458	−1.600 (−7.569, 2.934)	−0.403 (−3.156, 0.525)	0.650 (0.109, 2.863)
	Age and rs2076735	−0.749 (−4.400, 1.857)	−0.344 (−2.548, 0.730)	0.612 (−4.333, 5.005)
Thyroid function abnormality				
	Urinary iodine and rs2268458	0.552 (−0.328, 1.203)	0.718 (−0.432, 1.757)	0.294 (−1.923, 2.888)
	Urinary iodine and rs2076735	0.267 (−1.192, 0.940)	0.346 (−1.056, 1.723)	0.460 (−2.584, 4.086)
Subclinical hyperthyroidism				
	Urinary iodine and rs2268458	0.574 (−0.982, 1.432)	1.445 (−3.276, 10.132)	0.512 (−1.669, 2.644)
	Urinary iodine and rs2076735	0.525 (−3.384, 1.549)	0.885 (−2.821, 4.844)	0.437 (−2.341, 3.737)
	Thyroid disease in relatives and rs2268458	−1.237 (−9.542, 3.235)	−0.794 (−10.752, 1.212)	0.311 (−4.727, 6.154)

Note: Additive (*RERI/AP/S*) interaction of subclinical hypothyroidism and district of residence with the two genetic variants (rs2268458, rs2076735) were not estimable due to sparse data, and are therefore not shown. The interaction between subclinical hyperthyroidism and rs2076735 was likewise not estimable due to sparse data.

**Table 4:** Thyroid function abnormalities and subclinical thyroid dysfunction: multiplicative interaction of SNPs with influencing factors.

		Multiplicative Model		
		*p*	*β*	*PR* (95% *CI*)
FT_3_ abnormality				
	Sex × rs2268458	0.355	−1.069	0.343 (0.036, 3.304)
	Sex × rs2076735	0.878	0.185	1.203 (0.113, 12.759)
	Urinary iodine × rs2268458	0.096	0.007	1.007 (0.999,1.016)
	Urinary iodine × rs2076735	0.669	−0.002	0.998 (0.991, 1.006)
FT_4_ abnormality				
	Age × rs2268458	0.620	−0.341	0.711 (0.185, 2.736)
	Age × rs2076735	0.142	−1.576	0.207 (0.025,1.697)
Thyroid function abnormality				
	Urinary iodine × rs2268458	0.074	0.003	1.003 (0.9997, 1.006)
	Urinary iodine × rs2076735	0.867	0.0003	1.000 (0.997, 1.004)
	Urinary iodine × rs2268458	0.719	0.003	1.003 (0.988, 1.018)
	Urinary iodine × rs2076735	0.426	0.008	1.008 (0.989, 1.028)
	Thyroid disease in relatives × rs2268458	0.993	−0.012	0.988 (0.062, 15.819)

Note: Multiplicative-scale interaction of subclinical hypothyroidism and district of residence with the two genetic variants (rs2268458, rs2076735) were not estimable due to sparse data, and are therefore not shown. The interaction between subclinical hyperthyroidism and rs2076735 was likewise not estimable due to sparse data.

**Table 5:** Subgroup-Specific effects of influencing factors on thyroid function abnormality and subclinical thyroid dysfunction, stratified by genotypes.

Thyroid Outcomes	Influencing Factors	*p*	*PR* (95% *CI*)
Thyroid function abnormality			
*NKX2-1* rs2076735	TT + TC		
	Urinary Iodine		
	<100 μg/L	0.031	3.093 (1.108, 8.636)
	≥300 μg/L	0.172	2.231 (0.706, 7.046)
Subclinical hyperthyroidism			
*NKX2-1* rs2076735	CC		
	Drinking kelp and laver soup frequently	0.049	3.636 (1.006, 13.140)
	Thyroid disease in relatives	0.029	4.114 (1.152, 14.688)
*TSHR* rs2268458	TT		
	Drinking kelp and laver soup frequently	0.023	4.814 (1.245, 18.610)

Note: Reference: urinary iodine 100–299 μg/L.

## Data Availability

The data are not publicly available due to privacy. The raw data supporting the conclusions of this article will be made available upon request.

## Supplementary Materials

The following supporting information can be downloaded at: https://www.mdpi.com/article/10.3390/nu18183085/s1, Table S1: Distribution of external factors; Table S2: Potential factors identified by univariate Poisson regression (*p* < 0.1); Table S3: Multicollinearity diagnostics for variables in each multivariate Poisson regression model; Table S4: Genotyping quality control metrics and Hardy–Weinberg equilibrium test for the two SNPs; Table S5: Association of *TSHR* and *NKX2-1* SNPs with thyroid function abnormalities and subclinical thyroid dysfunction; Table S6: Event counts and model parameters for each genotype stratum; Table S7: Potential factors identified by univariate logistic regression analysis (*p* < 0.1); Table S8: Analysis of influencing factors for thyroid function abnormalities and subclinical thyroid dysfunction (multivariate adjusted logistic regression); Table S9: Subgroup-Specific effects of genotypes on associations of influencing factors with thyroid function abnormalities and subclinical thyroid dysfunction (logistic regression analysis).

## Abbreviations

The following abbreviations are used in this manuscript:

UIC	Urinary iodine concentration
HPT	Hypothalamic–pituitary–thyroid
*TSHR*	Thyroid stimulating hormone receptor
*NKX2-1*	NK2 homeobox 1
TTF-1	Thyroid transcription factor 1
*TPO*	Thyroid peroxidase
*TG*	Thyroglobulin
*DUOX2*	Dual oxidase 2
DNA	Deoxyribonucleic acid
SNPs	Single nucleotide polymorphisms
PCR	Polymerase chain reaction
TJCDC	Tianjin Centers for Disease Control and Prevention
BMI	Body mass index
UNICEF	United Nations Children’s Fund
IGN	Iodine Global Network
FT_3_	Free triiodothyronine
FT_4_	Free thyroxine
TSH	Thyroid-stimulating hormone
1st-PCRP	First PCR primer
2nd-PCRP	Second PCR primer
RCS	Restricted cubic spline
*OR*	Odds ratio
95% *CI*	95% confidence interval
GEE	Generalized estimating equations
AIC	Akaike Information Criterion
*PR*	Prevalence ratio
*RERI*	Relative excess risk due to interaction
*AP*	Attributable proportion
*S*	Synergy index
VIF	Variance inflation factor
TPOAb	Thyroid peroxidase antibody
TgAb	Thyroglobulin antibody
