# Supplementary material for: Iodine Nutrition, Non-Genetic Factors, and Two Candidate Polymorphisms Associated with Thyroid Function Abnormalities Among Children Aged 8–10 Years in Iodine-Adequate Areas of Tianjin, China
Source: Nutrients. 2026 Sep 20;18(18):3085. doi: 10.3390/nu18183085 (PMC13610479; doi:10.3390/nu18183085)
Supplement: Supplementary file 1 [file nutrients-18-03085-s001.zip › nutrients-4528514-supplementary.pdf]

Table S1 Distribution of external factors

Groups	n	Percent	Groups	n	Percent
Exposure time of electronic instrument			Psychological trauma		
<1h/day	582	58.38	No	949	95.19
≥1h/day	415	41.62	Yes	48	4.81
Dietary habits			Having a cold		
Primarily vegetarian/ Primarily meat-based	194	19.46	No	906	90.87
Balanced omnivorous diet	803	80.54	Yes	91	9.13
Eating seafood frequently			Radiographic examination of the head and neck		
No	855	85.76	No	919	92.18
Yes	142	14.24	Yes	78	7.82
Eating pickled food frequently			Iodine supplement/ drug use (past year)		
No	948	95.09	No	815	81.75
Yes	49	4.91	Yes	182	18.25
Drinking kelp and laver soup frequently			Dwelling house		
No	773	77.53	Single-story house	365	36.61
Yes	224	22.47	Multistory building	632	63.39
Dietary tastes			Noise in surroundings		
Overly salty	35	3.51	No/ Small	822	82.45
Moderately salty/ Light diet	962	96.49	Loud	175	17.55
Tea consumption			Factory within 30 m of residence		
No	964	96.69	No	972	97.49
Yes	33	3.31	Yes	25	2.51
Indoor insecticide use			Thyroid disease in relatives		
No	394	39.52	No	867	86.96
Occasional/ Often	603	60.48	Yes	130	13.04
Physical activity			Father's education		
< 3 times/ week	316	31.70	< high school	396	39.72
≥ 3 times/ week	681	68.30	≥ high school	601	60.28
Outdoor activity			Mother's education		
< 1h/day	633	63.49	< high school	409	41.02
≥ 1h/day	364	36.51	≥ high school	588	58.98

Table S1 (continued)

Groups	n	Percent	Groups	n	Percent
Pressure			Father having a stable job		
No/ Low	951	95.39	No	494	49.55
High	46	4.61	Yes	503	50.45
Anger			Mother having a stable job		
No/ Occasional	856	85.86	No	585	58.68
Often	141	14.14	Yes	412	41.32
Anxiety					
No/ Mild	959	96.19			
Serious	38	3.81			

Table S2 Potential factors identified by univariate Poisson regression (*P*<0.1)

Thyroid outcomes	Influencing factors	*P*	*PR*(95%*CI*)
FT_3 abnormality			
	Sex	0.035	1.601(1.035, 2.476)
	Urinary Iodine		
	<100 μg/L	0.091	1.479(0.940, 2.327)
	≥300 μg/L	0.868	0.942(0.467, 1.902)
	Father having a stable job	0.051	0.648(0.419, 1.002)
FT_4 abnormality			
	Age	0.065	1.486(0.976, 2.264)
	Indoor insecticide use	0.062	1.851(0.971, 3.531)
TSH abnormality			
	Father's education	0.089	1.537(0.937, 2.523)
Thyroid function abnormality			
	Urinary Iodine		
	<100 μg/L	0.064	1.323(0.984, 1.778)
	≥300 μg/L	0.334	1.217(0.817, 1.812)
	Iodine supplement/ drug use (past year)	0.072	1.366(0.972, 1.920)
	Psychological trauma	0.094	1.541(0.929, 2.557)
Subclinical hyperthyroidism			
	Urinary Iodine		
	<100 μg/L	0.069	1.943(0.951, 3.970)
	≥300 μg/L	0.265	1.705(0.667, 4.357)
	Drinking kelp and laver soup frequently	0.091	1.800(0.910, 3.561)
	Thyroid disease in relatives	0.026	2.309(1.107, 4.816)
	Father's education	0.044	2.224(1.021, 4.845)
Subclinical hypothyroidism			
	Psychological trauma	0.050	3.264(1.002, 10.636)
	Mother having a stable job	0.094	2.051(0.885, 4.753)

Note: For urinary iodine, 100–299 μg/L was designated as the reference group.

Table S3 Multicollinearity diagnostics for variables in each multivariate Poisson regression model

Thyroid outcomes	Influencing factors	Range of VIF
FT_3 abnormality	Age, Sex, BMI-z-score, Urinary Iodine, Recruitment year, district, Father having a stable job	1.003-1.047
FT_4 abnormality	Age, Sex, BMI-z-score, Urinary Iodine, Recruitment year, district, Indoor insecticide use	1.019-1.046
TSH abnormality	Age, Sex, BMI-z-score, Urinary Iodine, Recruitment year, district, Father's education	1.018-1.371
Thyroid function abnormality	Age, Sex, BMI-z-score, Urinary Iodine, Recruitment year, district, Iodine supplement/ drug use (past year), Psychological trauma	1.014-1.052
Subclinical hyperthyroidism	Age, Sex, BMI-z-score, Urinary Iodine, Recruitment year, district, Drinking kelp and laver soup frequently, Thyroid disease in relatives, Father's education	1.022-1.374
Subclinical hypothyroidism	Age, Sex, BMI-z-score, Urinary Iodine, Recruitment year, district, Psychological trauma, Mother having a stable job	1.006-1.052

Note: All VIF values were < 5, indicating no significant multicollinearity.

Table S4 Genotyping quality control metrics and Hardy-Weinberg equilibrium test for the two SNPs

	SNP	Call rate (subset)	MAF	HWE *P*	Genotype counts
TSHR	rs2268458	100%	0.269	0.576	TT: 201, CT: 142, CC: 29
NKX2-1	rs2076735	100%	0.164	0.449	TT: 8, TC: 106, CC: 258

Table S5 Association of *TSHR* and *NKX2-1* SNPs with thyroid function abnormalities and subclinical thyroid dysfunction

Gene region	SNP	Genotype	Number	Normal group	Disease group	χ^2	*P*
FT_3 abnormality							
TSHR	rs2268458	CC	29	28	1	0.167	1.000*
		CT	142	137	5		
		TT	201	193	8		
		TT+CT	343	330	13	<0.001	1.000**
		CC	29	28	1		
		CC+CT	171	165	6	0.057	1.000
		TT	201	193	8		
NKX2-1	rs2076735	CC	258	251	7	2.047	0.409*
		TC	106	100	6		
		TT	8	8	0		
		TT+TC	114	108	6	0.862	0.353**
		CC	258	251	7		
		CC+TC	364	351	13	——	1.000*
		TT	8	8	0		
FT_4 abnormality							
TSHR	rs2268458	CC	29	28	1	0.235	0.940*
		CT	142	133	9		
		TT	201	190	11		
		TT+CT	343	323	20	0.013	0.909**
		CC	29	28	1		
		CC+CT	171	161	10	0.024	0.876
		TT	201	190	11		
NKX2-1	rs2076735	CC	258	244	14	0.280	0.768*
		TC	106	99	7		
		TT	8	8	0		
		TT+TC	114	107	7	0.076	0.783
		CC	258	244	14		
		CC+TC	364	343	21	——	1.000*
		TT	8	8	0		
TSH abnormality							
TSHR	rs2268458	CC	29	25	4	2.680	0.245*
		CT	142	134	8		
		TT	201	184	17		
		TT+CT	343	318	25	0.799	0.371**
		CC	29	25	4		
		CC+CT	171	159	12	0.267	0.606
		TT	201	184	17		

Table S5 (continued)

Gene region	SNP	Genotype	Number	Normal group	Disease group	χ^2	P
NKX2-1	rs2076735	CC	258	236	22	0.375	0.830*
		TC	106	99	7		
		TT	8	8	0		
		TT+TC	114	107	7	0.627	0.429
		CC	258	236	22		
		CC+TC	364	335	29	——	1.000*
		TT	8	8	0		
Thyroid function abnormality							
TSHR	rs2268458	CC	29	23	6	0.840	0.645*
		CT	142	121	21		
		TT	201	167	34		
		TT+CT	343	288	55	0.151	0.697
		CC	29	23	6		
		CC+CT	171	144	27	0.085	0.770
		TT	201	167	34		
NKX2-1	rs2076735	CC	258	216	42	1.017	0.601*
		TC	106	88	18		
		TT	8	8	0		
		TT+TC	114	96	18	0.014	0.906
		CC	258	216	42		
		CC+TC	364	304	60	0.590	0.442**
		TT	8	8	0		
Subclinical hyperthyroidism							
TSHR	rs2268458	CC	29	27	2	5.854	0.043*
		CT	142	141	1		
		TT	201	192	9		
		TT+CT	343	333	10	——	0.238*
		CC	29	27	2		
		CC+CT	171	168	3	2.195	0.138
		TT	201	192	9		
NKX2-1	rs2076735	CC	258	249	9	0.194	1.000*
		TC	106	103	3		
		TT	8	8	0		
		TT+TC	114	111	3	0.013	0.910**
		CC	258	249	9		
		CC+TC	364	352	12	——	1.000*
		TT	8	8	0		

Table S5 (continued)

Gene region	SNP	Genotype	Number	Normal group	Disease group	χ^2	P
Subclinical hypothyroidism							
TSHR	rs2268458	CC	29	27	2	1.376	0.446*
		CT	142	135	7		
		TT	201	194	7		
		TT+CT	343	329	14	0.058	0.810**
		CC	29	27	2		
		CC+CT	171	162	9	0.712	0.399
		TT	201	194	7		
NKX2-1	rs2076735	CC	258	246	12	0.120	1.000*
		TC	106	102	4		
		TT	8	8	0		
		TT+TC	114	110	4	0.050	0.823**
		CC	258	246	12		
		CC+TC	364	348	16	——	1.000*
		TT	8	8	0		

Note: *The Fisher's exact test was used; **The chi-square test with continuity correction was used.

Table S6 Event counts and model parameters for each genotype stratum

Genotype stratum	Outcome	Exposure of interest	Events (n)	Total (n)	Model parameters	Events per parameter
NKX2-1 rs2076735 TT+TC	Thyroid function abnormality	UIC	18	114	3	6.00
TSHR rs2268458 TT	Subclinical hyperthyroidism	Kelp/laver soup consumption	9	201	4	2.25
NKX2-1 rs2076735 CC	Subclinical hyperthyroidism	Kelp/laver soup consumption	9	258	4	2.25
NKX2-1 rs2076735 CC	Subclinical hyperthyroidism	Family history of thyroid disease	9	258	4	2.25

Table S7 Potential factors identified by univariate logistic regression analysis ($P < 0.1$)

Thyroid outcoms	Influencing factors	*P*	*OR*(95%*CI*)
FT_3 abnormality			
	Sex	0.034	1.665(1.039,2.668)
	Urinary Iodine		
	<100 μg/L	0.092	1.533(0.933,2.519)
	≥300 μg/L	0.868	0.938(0.442,1.990)
	Father having a stable job	0.050	0.625(0.390,1.001)
FT_4 abnormality			
	Age	0.069	1.514(0.968,2.369)
	Indoor insecticide use	0.060	1.902(0.973,3.720)
TSH abnormality			
	Father's education	0.087	1.585(0.935,2.687)
Thyroid function abnormality			
	Urinary Iodine		
	<100 μg/L	0.066	1.405(0.978,2.018)
	≥300 μg/L	0.339	1.267(0.780,2.057)
	Iodine supplement/ drug use (past year)	0.080	1.473(0.955,2.272)
Subclinical hyperthyroidism			
	Urinary Iodine		
	<100 μg/L	0.069	1.991(0.948,4.183)
	≥300 μg/L	0.267	1.737(0.655,4.605)
	Drinking kelp and laver soup frequently	0.093	1.846(0.903,3.771)
	Thyroid disease in relatives	0.028	2.406(1.101,5.257)
	Father's education	0.043	2.281(1.026,5.074)
Subclinical hypothyroidism			
	Psychological trauma	0.055	3.422(0.975,12.010)
	Mother having a stable job	0.094	2.085(0.883,4.925)

Note: For urinary iodine, 100–299 μg/L was designated as the reference group.

Table S8 Analysis of influencing factors for thyroid function abnormalities and subclinical thyroid dysfunction (multivariate adjusted logistic regression)

Thyroid outcoms	Influencing factors	*P*	*OR*(95%*CI*)
FT_3 abnormality			
	Sex	0.026	1.658(1.061, 2.592)
	Urinary Iodine		
	<100 μg/L	0.047	1.679(1.007, 2.799)
	≥300 μg/L	0.977	0.990(0.490, 1.999)
FT_4 abnormality			
	Age	0.047	1.458(1.005, 2.114)
Thyroid function abnormality			
	Urinary Iodine		
	<100 μg/L	0.012	1.421(1.080, 1.868)
	≥300 μg/L	0.144	1.291(0.917,1.818)
Subclinical hyperthyroidism			
	Urinary Iodine		
	<100 μg/L	0.197	1.658(0.769, 3.577)
	≥300 μg/L	0.047	1.946(1.008, 3.756)
	Thyroid disease in relatives	0.018	2.260(1.147, 4.454)
Subclinical hypothyroidism			
	District of residence	0.009	1.202(1.046, 1.381)

Note: For urinary iodine, 100–299 μg/L was designated as the reference group.

Table S9 Subgroup-Specific effects of genotypes on associations of influencing factors with thyroid function abnormalities and subclinical thyroid dysfunction (logistic regression analysis)

Thyroid outcoms	Influencing factors	*P*	*OR*(95%*CI*)
Thyroid function abnormality			
NKX2-1 rs2076735	TT+TC		
	Urinary Iodine		
	<100 μg/L	0.031	3.855(1.135,13.094)
	≥300 μg/L	0.175	2.524(0.662,9.626)
Subclinical hyperthyroidism			
NKX2-1 rs2076735	CC		
	Drinking kelp and laver soup frequently	0.049	3.852(1.003,14.803)
	Thyroid disease in relatives	0.032	4.442(1.141,17.297)
TSHR rs2268458	TT		
	Drinking kelp and laver soup frequently	0.022	5.245(1.266,21.734)
